# FACING TRUSTWORTHINESS: AGE-GROUP DIFFERENCES IN TRUST-RELATED DECISION-MAKING AND LEARNING

**DOI:** 10.1093/geroni/igad104.3341

**Published:** 2023-12-21

**Authors:** Marilyn Horta, Alayna Shoenfelt, Nichole Lighthall, Eliany Perez, Ian Frazier, Tian Lin, Amber Heemskerk, Natalie Ebner

**Affiliations:** University of Florida, Gainesville, Florida, United States; University of Florida, Gainesville, Florida, United States; University of Central Florida, Orlando, Florida, United States; University of Florida, Gainesville, Florida, United States; Rutgers University, New Brunswick, New Jersey, United States; University of Florida, Gainesville, Florida, United States; University of Florida, Gainesville, Florida, United States; University of Florida, Gainesville, Florida, United States

## Abstract

Facial impressions contribute to evaluations of trustworthiness. Older adults are especially vulnerable to trust violations, incurring risks for deception and exploitation. Using the newly developed Social Iowa Gambling Task (S-IGT), we examined age-group differences in the impact of facial trustworthiness on decision-making. Advantageous decks were represented by trustworthy (congruent condition, CS-IGT) or untrustworthy (incongruent condition, IS-IGT) faces and disadvantageous decks were represented by untrustworthy faces in the CS-IGT and by trustworthy faces in the IS-IGT. Younger (n = 143) and older (n = 129) participants completed either the standard IGT, CS-IGT, or IS-IGT. Both age groups preferred trustworthy faces in their initial choices. Older adults performed worse than younger adults across all tasks over time. Further, compared to younger adults, older adults performed worse on the IS-IGT, suggesting that incongruent facial cues interfered with older adults’ performance, which aligns with reduced sensitivity to negative social reputations in aging. Exploratory multilevel modeling indicated that age-group differences were most pronounced in the last 40 trials across all tasks. Together these findings indicate that differences between younger and older adults in experience-dependent decision-making are magnified in social contexts that involve a “wolf in sheep’s clothing,” which may reflect age-related difficulties in information integration.

